# Supplementary material for: Programming actuation onset of a liquid crystalline elastomer via isomerization of network topology
Source: Nat Commun. 2023 Oct 26;14:6822. doi: 10.1038/s41467-023-42594-8 (PMC10603074; doi:10.1038/s41467-023-42594-8)
Supplement: Supplementary file 1 — Supplementary Information [file 41467_2023_42594_MOESM1_ESM.pdf]

Supplementary information

Programming actuation onset of a liquid crystalline elastomer via isomerization of network topology

Guancong Chen[1,2], Haijun Feng[2], Xiaorui Zhou[2], Feng Gao[3], Kai Zhou[2], Youju Huang[1], Binjie Jin[2]*, Tao Xie[2], Qian Zhao[2]*

[1]College of Material, Chemistry and Chemical Engineering, Key Laboratory of Organosilicon Chemistry and Material Technology, Ministry of Education, Hangzhou Normal University, Hangzhou 311121, Zhejiang, China.

[2]State Key Laboratory of Chemical Engineering, College of Chemical and Biological Engineering, Zhejiang University, Hangzhou 310027, Zhejiang, China.

[3]National Engineering Laboratory for Textile Fiber Materials & Processing Technology, Zhejiang Sci-Tech University, Hangzhou 310018, Zhejiang, China.

*Correspondence to: binjie_jin@zju.edu.cn; qianzhao@zju.edu.cn.

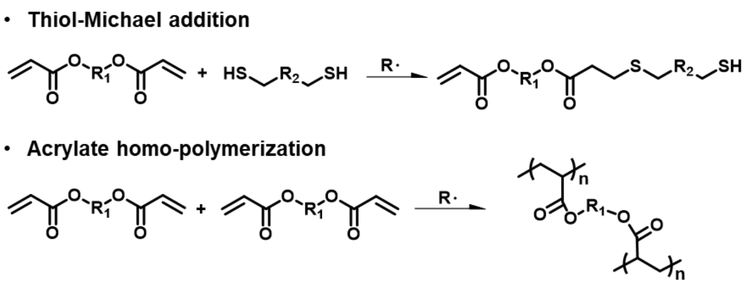

Supplementary Fig. 1 Two reactions initiated for network synthesis.

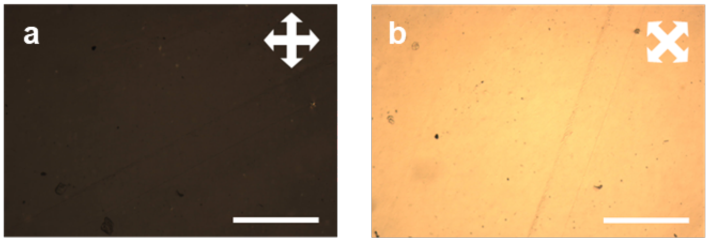

Supplementary Fig. 2 Polarized optical microscopy (POM) images of the programmed LCE (pre-stretched strain: 50%, T_p = 120 °C, t_p = 15 min) under cross polarizer at (**a**) 0° (dark) and (**b**) 45° (light), respectively. Scale bar: 500 μm.

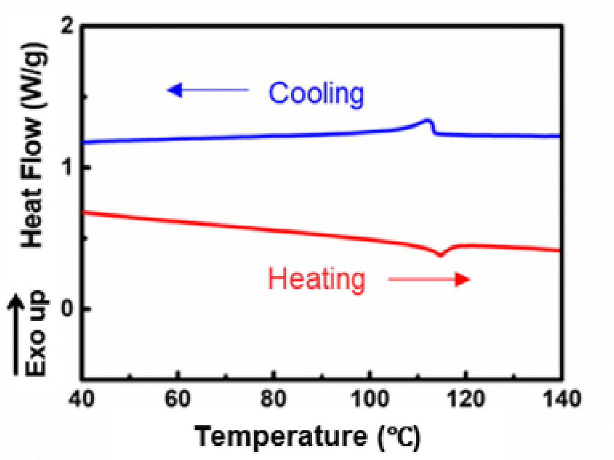

Supplementary Fig. 3 Differential scanning calorimetry (DSC) curve of the pristine LCE network.

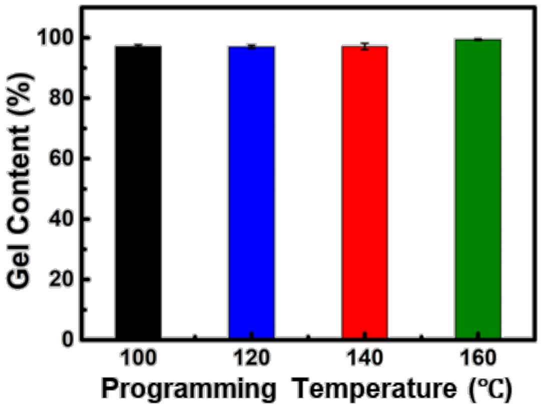

Supplementary Fig. 4 Gel content of the LCEs programmed at different temperatures (t_p = 10 min). The gel content is calculated as follows: gel content (%) = $M/M_0 \times 100\%$, where the M and M_0 are the remaining mass after swelling in toluene for 24 hours and initial mass, respectively. Error bars represent standard deviation, n=5.

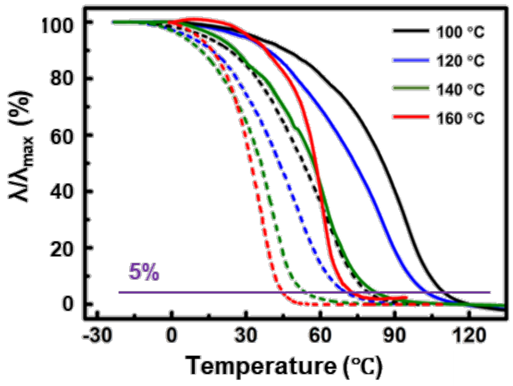

Supplementary Fig. 5 Correlation between the normalized actuation strain and temperature. The LCE samples were programmed at different temperatures for 10 minutes with the same pre-stretched strain (50%). The solid and dash curves represent the heating and cooling actuation, respectively.

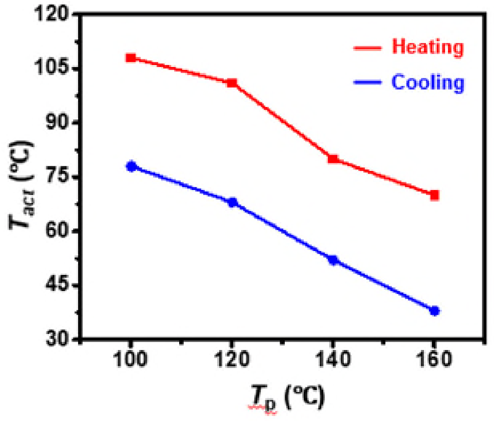

Supplementary Fig. 6 Correlation between T_{act} and T_p. The T_{act}s are deduced from Figure S5.

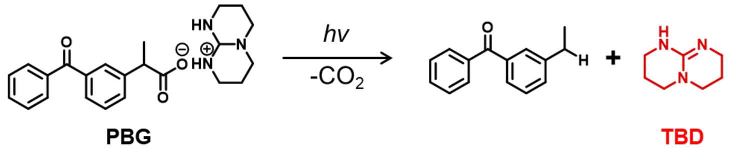

Supplementary Fig. 7 UV-initiated releasing reaction of TBD.

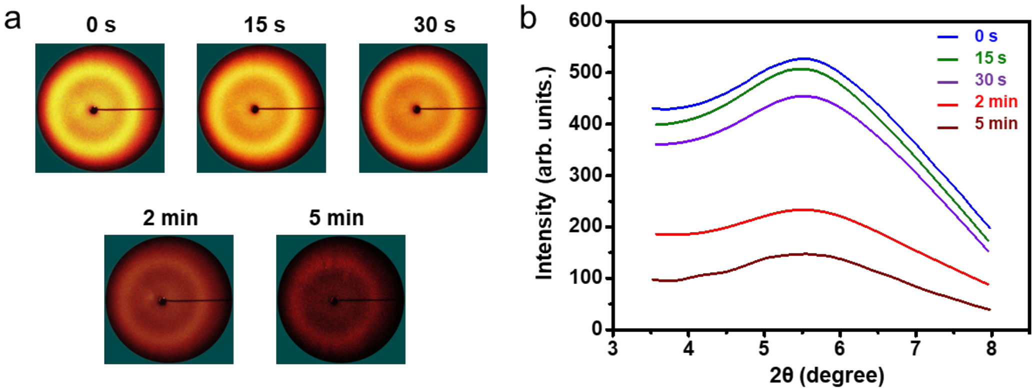

Supplementary Fig. 8 X-ray characterization of the LCEs (**a**) 2D-WAXD images of LCEs with different irradiation times (T_p = 120 °C, t_p = 10 min). (**b**) 1D-X-ray data derived from (**a**).

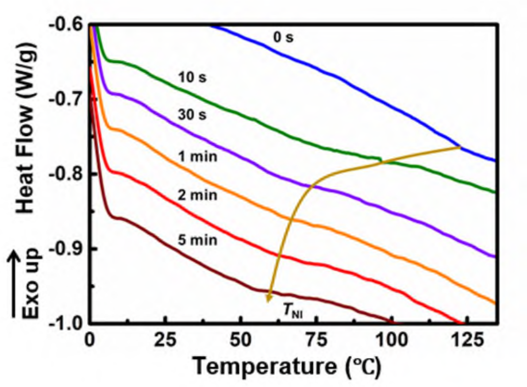

Supplementary Fig. 9 DSC curves of the programmed LCEs with different irradiation times (T_p = 120 °C, t_p = 10 min).

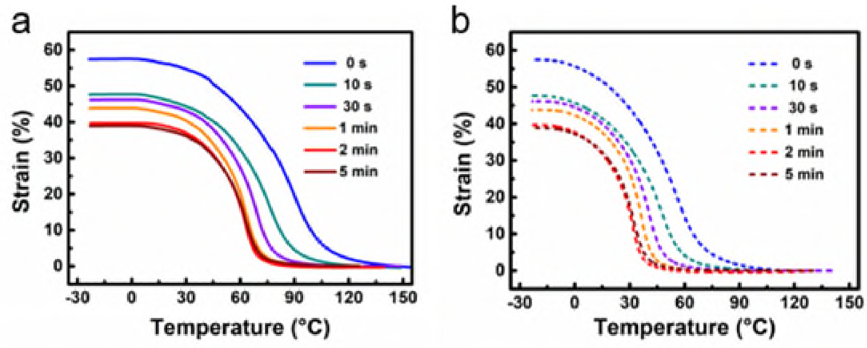

Supplementary Fig. 10 Correlation between the actuation strain of the samples (irradiated for different times) and temperature (pre-stretched strain: 50%, T_p: 120 ºC, t_p: 10 min). **(a)** heating cycle. **(b)** cooling cycle.

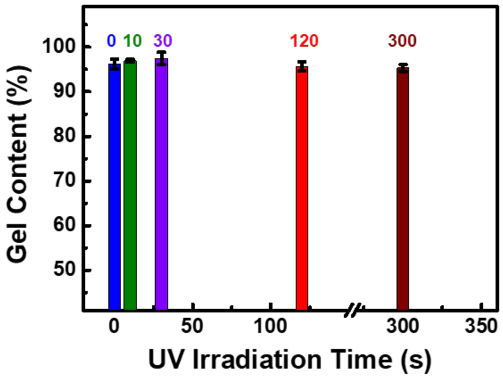

Supplementary Fig. 11 Gel fraction of the LCEs with different irradiation times (T_p = 120 °C, t_p = 10 min). Error bars represent standard deviation, n=5.

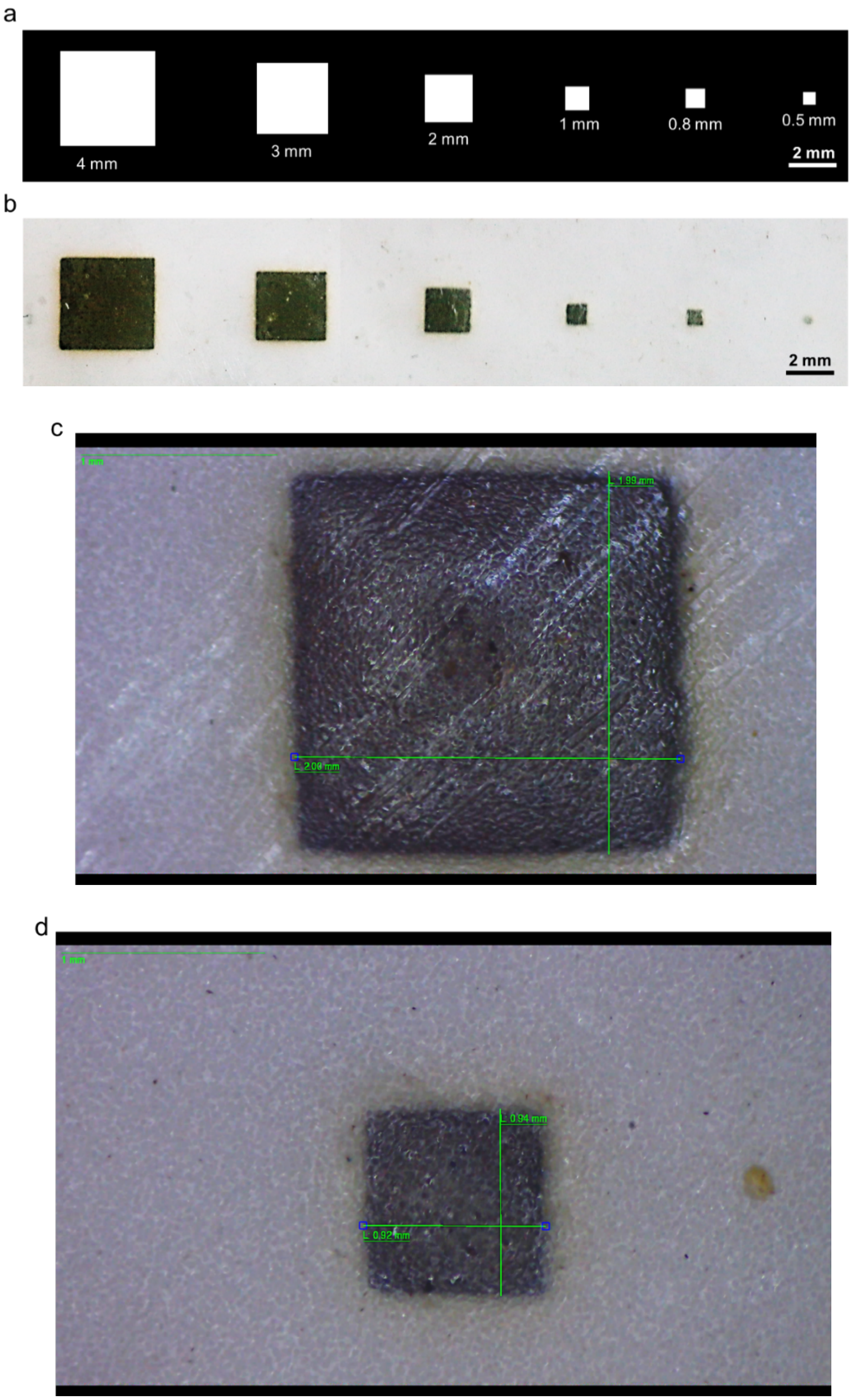
a
4 mm
3 mm
2 mm
1 mm
0.8 mm
0.5 mm
2 mm
b
2 mm
c
d

e

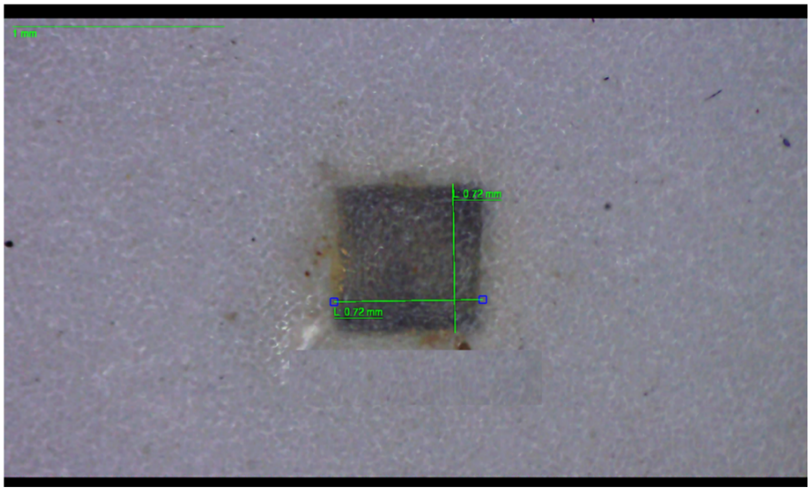

f

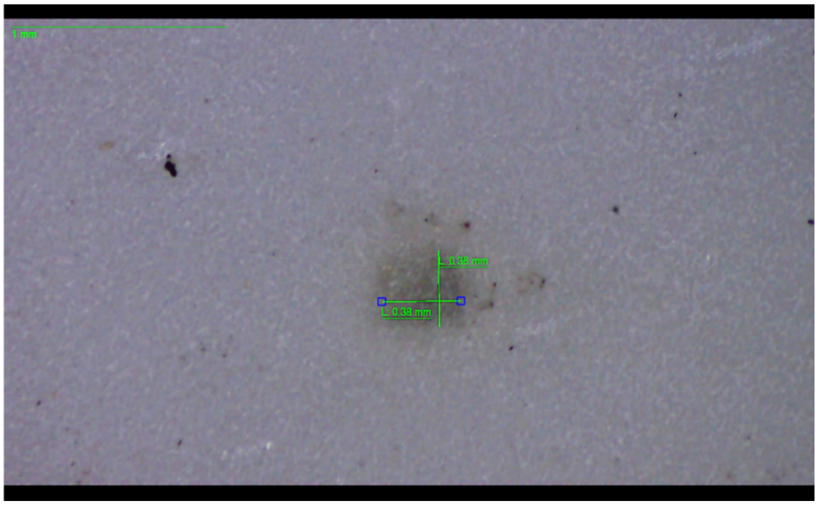

Supplementary Fig. 12 Resolution characterization of the light-definable T_{NI}. (**a)** Scheme of the photo mask. The white squares represent the irradiation regions, with the length labeled (mm). (**b)** Visual photos of the UV-irradiated LCEs. The black areas are the irradiated regions. Individual photos of the irradiated areas corresponding to the square with a length of (**c)** 2 mm, (**d)** 1 mm, (**e)** 0.8 mm, and (**f)** 0.5 mm, respectively.

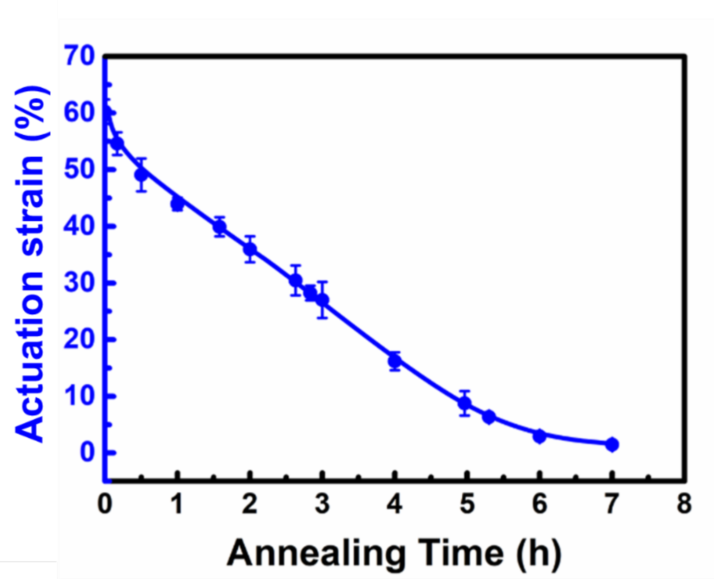

Supplementary Fig. 13 Relationship between the residual actuation strain and anneal time (annealing temperature: 120 ℃, original actuation strain: 60%). Error bars represent standard deviation, n=5.

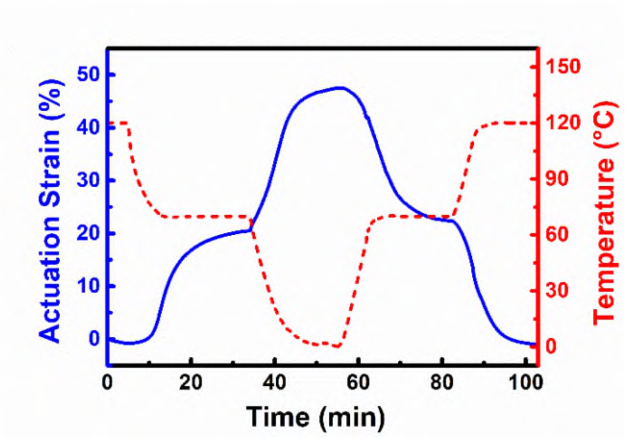

Supplementary Fig. 14 DMA curve of the sequential actuation of the LCE with two T_{NI}s.

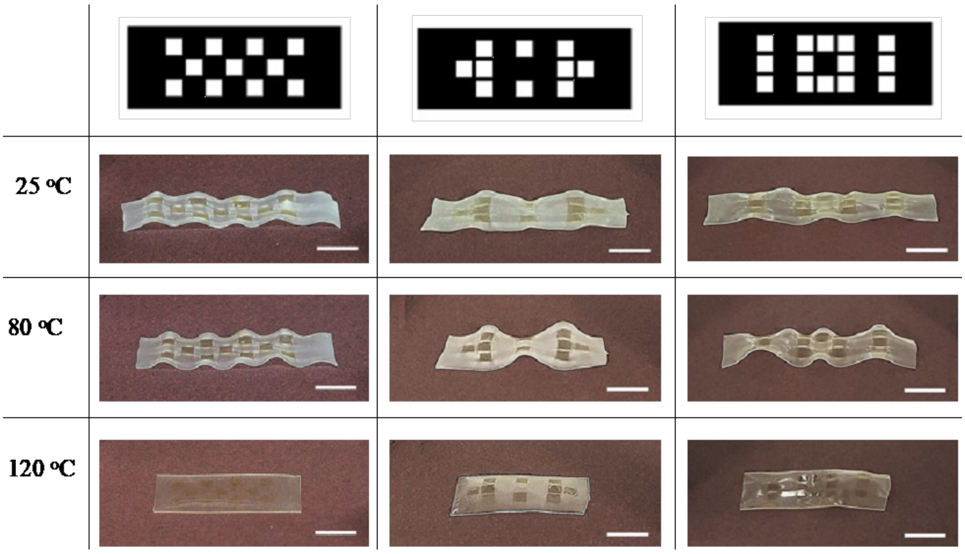

Supplementary Fig. 15 Active LCE structures when two different T_{NI}s embedded in a pixelated manner. The white areas represent the UV-exposed region (irradiated for 2 mins). Scale bar: 1 cm.

Supplementary Movie 1: Active LCE pyramid with two distinct T_{NI}s.
Supplementary Movie 2: Active LCE gripper.
Supplementary Movie 3: Self-locked LCE four-leaf clover (single T_{NI}).
Supplementary Movie 4: Self-correctable LCE four-leaf clover (quadri T_{NI}s)
