# Supplementary material for: Programming actuation onset of a liquid crystalline elastomer via isomerization of network topology
Source: Nat Commun. 2023 Oct 26;14:6822. doi: 10.1038/s41467-023-42594-8 (PMC10603074; doi:10.1038/s41467-023-42594-8)
Supplement: Supplementary file 3 — Description of Additional Supplementary Files [file 41467_2023_42594_MOESM3_ESM.pdf]

Description of Additional Supplementary Files

File Name: Supplementary Movie 1
Description: Active LCE pyramid with two distinct T_{NI}s.

File Name: Supplementary Movie 2
Description: Active LCE gripper.

File Name: Supplementary Movie 3
Description: Self-locked LCE four-leaf clover (single T_{NI}).

File Name: Supplementary Movie 4
Description: Self-correctable LCE four-leaf clover (quadri T_{NI}s)
